# High-Order Spatial Simulation Using Legendre-Like Orthogonal Splines

**DOI:** 10.1007/s11004-018-9741-2

**Published:** 2018-05-17

**Authors:** Ilnur Minniakhmetov, Roussos Dimitrakopoulos, Marcelo Godoy

**Affiliations:** 1 COSMO—Stochastic Mine Planning Laboratory, McGill University, Montreal, QC H3A 0E8 Canada; 2 Newmont Mining Corporation, Denver, CO USA

**Keywords:** Stochastic simulation, Orthogonal splines, High-order spatial statistics, Non-Gaussian distribution, Spatial complexity

## Abstract

High-order sequential simulation techniques for complex non-Gaussian spatially distributed variables have been developed over the last few years. The high-order simulation approach does not require any transformation of initial data and makes no assumptions about any probability distribution function, while it introduces complex spatial relations to the simulated realizations via high-order spatial statistics. This paper presents a new extension where a conditional probability density function (cpdf) is approximated using Legendre-like orthogonal splines. The coefficients of spline approximation are estimated using high-order spatial statistics inferred from the available sample data, additionally complemented by a training image. The advantages of using orthogonal splines with respect to the previously used Legendre polynomials include their ability to better approximate a multidimensional probability density function, reproduce the high-order spatial statistics, and provide a generalization of high-order simulations using Legendre polynomials. The performance of the new method is first tested with a completely known image and compared to both the high-order simulation approach using Legendre polynomials and the conventional sequential Gaussian simulation method. Then, an application in a gold deposit demonstrates the advantages of the proposed method in terms of the reproduction of histograms, variograms, and high-order spatial statistics, including connectivity measures. The C++ course code of the high-order simulation implementation presented herein, along with an example demonstrating its utilization, are provided online as supplementary material.

**Electronic supplementary material:**

The online version of this article (10.1007/s11004-018-9741-2) contains supplementary material, which is available to authorized users.

## Introduction

Geostatistical simulations are used to quantify the uncertainty of spatially distributed attributes of interest describing mineral deposits, petroleum reservoirs, hydrogeological horizons, environmental contaminants, and other spatially variant natural phenomena. Since the 1990s, multiple-point spatial simulation (MPS) methods and variations (Guardiano and Srivastava 1993; Strebelle 2002; Journel 2005, 2018; Zhang et al. 2006; Arpat and Caers 2007; Chugunova and Hu 2008; de Vries et al. 2009; Mariethoz and Renard 2010; Mariethoz et al. 2010; Straubhaar et al. 2011; De Iaco and Maggio 2011; Honarkhah 2011; Strebelle and Cavelius 2014; Chatterjee et al. 2012; Lochbühler et al. 2014; Mustapha et al. 2014; Rezaee et al. 2013; Toftaker and Tjelmeland 2013; Zhang et al. 2017; others) have been developed to advance the simulation methods beyond the past generation of second-order spatial statistics, which were typically based on Gaussian processes (e.g., Journel and Huijbregts 1978; David 1988; Goovaerts 1998; Chilès and Delfiner 1999). A limitation of MPS approaches is that they are largely algorithmic and do not consistently account for the high-order spatial relations in the available sample data. Patterns and complex spatial relations are derived from the so-termed training images (TIs), or geological analogues, rather than from sample data; this is a critical topic for relatively data-rich type applications, where data statistics have been shown to not be reproduced by simulated realizations based on MPS methods (Osterholt and Dimitrakopoulos 2018; Goodfellow et al. 2012). To address some of these limits, high-order simulation techniques for complex and non-Gaussian spatially distributed variables have also been developed (Mustapha and Dimitrakopoulos 2010, 2011; Mustapha et al. 2011; Tamayo-Mas et al. 2016; Minniakhmetov and Dimitrakopoulos 2017a), based on generating conditional distributions through Legendre polynomials (Lebedev 1965) and high-order spatial cumulants. Yao et al. (2018) developed a new computational model to significantly reduce the computation cost of the method. The high-order simulation approach does not require any transformation of initial data and makes no assumptions about the related probability distribution function. The approach reproduces high-order spatial statistics of sample data and a training image. The high-order spatial statistics are shown to capture directional multiple-point periodicity, connectivity of extreme values, and complex spatial architecture (Dimitrakopoulos et al. 2010). However, polynomial approximations do not always converge to analytic functions (Runge 1901; Boyd and Ong 2009; Fornberg and Zuev 2007). In addition, the high-order polynomials are very sensitive to rounding errors for values near the endpoints of an approximation domain; therefore, even if the interpolants converge in theory, they will diverge rapidly when computed (Platte et al. 2011). This is critical for the simulation of extreme values, as they are located at the endpoints of an approximation domain. In an effort to improve upon the limitations of polynomial approximation, a spline approximation of complex multidimensional functions is considered here (Piegl 1989; Hughes et al. 2005; Ruiu et al. 2016).

Splines are piecewise-defined polynomial functions in which pieces are connected by some condition of smoothness. The places where these pieces meet are called knots, and two adjacent knots form a knot interval, hereafter referenced simply as an interval. Knot locations have a significant impact on the quality and flexibility of approximation, particularly in the approximation of functions with discontinuities (López de Silanes et al. 2001; Sinha and Schunck 1992) and functions with locally high gradients (Malagù et al. 2014). Furthermore, through the proper choosing of the knot sequence, splines can accurately approximate very complex functions, such as the shapes of three-dimensional objects in a computer-aided geometric design (Hoschek and Lasser 1993; Park and Lee 2007). Therefore, splines are chosen herein to approximate complex multidimensional joint distributions. The most commonly used mathematical formulation in different applications of splines are B-splines (from basis spline). The construction of B-splines is straightforward and simple to implement; however, the high-order simulation framework proposed by Mustapha and Dimitrakopoulos (2010) assumes the orthogonality of basis functions, and, therefore, splines in the form of B-splines are not suitable for a high-order spatial simulation approach.

In this paper, Legendre-like splines (Wei et al. 2013) are used, which are shown to be orthogonal and can be easily integrated in the high-order simulation framework. There are two user-defined parameters used for the constructing of Legendre-like splines: the order of splines and the maximum number of knots. In practice, cubic splines (order 3) are commonly used (Hughes et al. 2005; Piegl 1989), as they provide efficient smooth approximation. For the cubic splines, the first four Legendre-like splines are defined at two endpoint knots of the approximation domain and Legendre polynomials up to order 3. Next, Legendre-like splines are constructed by adding an additional knot per Legendre-like spline until the user-defined maximum number of knots is reached. Increasing the number of knots improves the approximation and describes more complex relations in the available data in the same way that the high-order polynomials capture the complex behavior of the function to be approximated. Thus, the maximum number of knots reflects the maximum order of high-order spatial relations that can be calculated from the available data. This spline approach aims to improve the estimation of the conditional probability density function (cpdf) and overcome the limitations of polynomial approximations. In addition, the proposed approach provides a general framework for high-order simulation techniques. For example, by using only one interval for spline construction, the technique becomes the one proposed by Mustapha and Dimitrakopoulos (2010, 2011).

The paper is organized as follows. First, the high-order simulation framework is outlined. Then, two systems of basis functions are outlined: Legendre polynomials (Lebedev 1965) and Legendre-like orthogonal splines. In the following section, the capabilities of both systems are compared using a fully known dataset to demonstrate the advantages of orthogonal splines in simulating connected high values. Next, the proposed approach is applied to a gold deposit and compared with the sequential Gaussian simulation approach in terms of the reproduction of histograms, variograms, high-order spatial statistics, and the connectivity of high values. Discussion and conclusions follow. Supplementary material available online provides the C++ course code of the high-order sequential simulation implementation detailed in Sect. 2.

## Sequential High-Order Simulation

Let *Z*(**u**_*i*_) be a stationary ergodic random field indexed in *R*^*n*^, where ${\mathbf{u}}_{i} \in D \subseteq R^{n} (n = 1,2,3),i = 1 \ldots N$ and where *N* is the number of points in a discrete grid $D \subseteq R^{n}$. Random variables indexed on the grid $D \subseteq R^{n}$ are denoted by $Z_{i} \equiv Z({\mathbf{u}}_{i} )$, whereas their outcomes are denoted by $z_{i} = z({\mathbf{u}}_{i} )$. The focus of high-order simulation techniques is to simulate the realization of the random field $Z({\mathbf{u}}_{i} )$ for all nodes of a grid *D* with a given set of conditioning data ${\mathbf{d}}_{n} = \{ z({\mathbf{u}}_{\alpha } ),\alpha = 1 \ldots n\}$.

The joint probability density function $f({\mathbf{u}}_{0} ,{\mathbf{u}}_{1} , \ldots {\mathbf{u}}_{N} ;z_{0} ,z_{1} , \ldots z_{N} |{\mathbf{d}}_{n} )$ of the random field $Z({\mathbf{u}}_{i} )$ can be decomposed into the product of conditional univariate distributions using the basic concept of sequential simulation (Journel and Alabert 1989; Journel 1994; Dimitrakopoulos and Luo 2004)

1 $$ \begin{aligned} & f({\mathbf{u}}_{1} , \ldots {\mathbf{u}}_{N} ;z_{1} , \ldots z_{N} |{\mathbf{d}}_{{\mathbf{n}}} ) \\ &\quad = f({\mathbf{u}}_{2} , \ldots {\mathbf{u}}_{N} ;z_{2} , \ldots z_{N} |z_{1} ,{\mathbf{d}}_{{\mathbf{n}}} )f({\mathbf{u}}_{1} ;z_{1} |{\mathbf{d}}_{{\mathbf{n}}} ) \\ &\quad  = f({\mathbf{u}}_{3} , \ldots {\mathbf{u}}_{N} ;z_{3} , \ldots z_{N} |z_{1} ,z_{2} ,{\mathbf{d}}_{{\mathbf{n}}} )f({\mathbf{u}}_{2} ;z_{2} |z_{1} ,{\mathbf{d}}_{{\mathbf{n}}} )f({\mathbf{u}}_{1} ;z_{1} |{\mathbf{d}}_{{\mathbf{n}}} ) \\ &\quad  = \prod\limits_{i = 2}^{N} {f({\mathbf{u}}_{i} ;z_{i} |z_{1} , \ldots ,z_{i - 1} ,{\mathbf{d}}_{{\mathbf{n}}} )} f({\mathbf{u}}_{1} ;z_{1} |{\mathbf{d}}_{{\mathbf{n}}} ). \\ \end{aligned} $$

Accordingly, the random path of visiting all grid nodes is defined first. Then, starting from the first node in the random path, the value *z*_*i*_ is simulated based on the estimated cpdf $f({\mathbf{u}}_{i} ;z_{i} |z_{1} , \ldots ,z_{i - 1} ,{\mathbf{d}}_{{\mathbf{n}}} )$. Finally, the simulated value is added to the set of conditional data, and the process is repeated until all grid nodes in the random path are visited. Eventually, any resulting simulation represents a realization of the complex joint distribution $f({\mathbf{u}}_{0} ,{\mathbf{u}}_{1} , \ldots {\mathbf{u}}_{N} ;z_{0} ,z_{1} , \ldots z_{N} |{\mathbf{d}}_{{\mathbf{n}}} )$.

Without loss of generality, let **u**_0_ be the first node in the random path. According to Bayes’ rule (Lee 2012)

2 $$ f({\mathbf{u}}_{0} ;z_{0} |{\mathbf{d}}_{{\mathbf{n}}} ) = \frac{{f({\mathbf{u}}_{0} ,{\mathbf{u}}_{1} , \ldots ,{\mathbf{u}}_{n} ;z_{0} ,{\mathbf{d}}_{{\mathbf{n}}} )}}{{f({\mathbf{u}}_{1} , \ldots ,{\mathbf{u}}_{n} ;{\mathbf{d}}_{{\mathbf{n}}} )}}, $$

where $f({\mathbf{u}}_{0} ,{\mathbf{u}}_{1} , \ldots ,{\mathbf{u}}_{n} ;z_{0} ,{\mathbf{d}}_{{\mathbf{n}}} )$ is a joint probability density function and $f({\mathbf{u}}_{1} , \ldots ,{\mathbf{u}}_{n} ;{\mathbf{d}}_{{\mathbf{n}}} )$ can be calculated as

3 $$ f({\mathbf{u}}_{1} , \ldots ,{\mathbf{u}}_{n} ;{\mathbf{d}}_{{\mathbf{n}}} ) = \int {f({\mathbf{u}}_{0} ,{\mathbf{u}}_{1} , \ldots ,{\mathbf{u}}_{n} ;\xi_{0} ,{\mathbf{d}}_{{\mathbf{n}}} )} d\xi_{0}. $$

In this paper, the joint probability density function $f({\mathbf{u}}_{0} ,{\mathbf{u}}_{1} , \ldots ,{\mathbf{u}}_{n} ;z_{0} ,{\mathbf{d}}_{{\mathbf{n}}} )$ is approximated using Legendre polynomials and Legendre-like orthogonal splines.

### Approximation of a Joint Probability Density Using Orthogonal Functions

Let *f*(*z*) be a probability density function of a random variable *Z* defined on [*a*, *b*] and let $\varphi_{1} (z),\varphi_{2} (z), \ldots$ be a complete system of orthogonal functions in [*a*, *b*], then *f*(*z*) can be approximated by the finite number *ω* of functions $\varphi_{1} (z),\varphi_{2} (z), \ldots \varphi_{\omega } (z)$

4 $$ f(z) \approx \sum\limits_{m = 0}^{\omega } {L_{m} \varphi_{m} (z)}, $$

where *L*_*m*_ are coefficients of approximation. The system of functions $\varphi_{1} (z),\varphi_{2} (z), \ldots \varphi_{\omega } (z)$ is orthogonal

5 $$ \int\limits_{a}^{b} {\varphi_{k} \varphi_{m} (z){\text{d}}z} = \delta_{km}, $$

where $$ \delta_{mk} = \left\{ {\begin{array}{*{20}l} {1,} \hfill & {m = k} \hfill \\ {0,} \hfill & {m \ne k} \hfill \\ \end{array} } \right. $$ is the Kronecker delta, and, therefore, $\forall k = 0 \ldots \omega$

6 $$ \int\limits_{a}^{b} {\varphi_{k} (z)f(z){\text{d}}z} \approx \int\limits_{a}^{b} {\varphi_{k} \sum\limits_{m = 0}^{\omega } {L_{m} \varphi_{m} (z)} {\text{d}}z} = \sum\limits_{m = 0}^{\omega } {L_{m} } \int\limits_{a}^{b} {\varphi_{k} \varphi_{m} (z){\text{d}}z} = \sum\limits_{m = 0}^{\omega } {L_{m} } \delta_{mk} = L_{k}. $$

By definition

7 $$ E[\varphi_{k} (z)] = \int\limits_{a}^{b} {\varphi_{k} (z)f(z){\text{d}}z}, $$

where *E* stands for mathematical expectation. The coefficients *L*_*m*_ can be estimated from the available data. Similarly, a joint probability density function $f(z_{0} ,z_{1} , \ldots z_{n} )$ of a set of random variables $Z_{0} ,Z_{1} , \ldots Z_{n}$ defined on $[a,b] \times [a,b] \times \ldots [a,b]$ can be approximated as

8 $$ f(z_{0} ,z_{1} , \ldots z_{n} ) \approx \sum\limits_{{m_{0} = 0}}^{{\omega_{0} }} {\sum\limits_{{m_{1} = 0}}^{{\omega_{1} }} { \cdots \sum\limits_{{m_{n} = 0}}^{{\omega_{n} }} {L_{{m_{0} ,m_{1} , \ldots ,m_{n} }} \varphi_{{m_{0} }} (z_{0} )\varphi_{{m_{2} }} (z_{1} ) \cdots \varphi_{{m_{n} }} (z_{n} )} } }. $$

Coefficients $L_{{m_{0} ,m_{1} , \ldots ,m_{n} }}$ are obtained from the orthogonality property

9 $$ \begin{aligned} & \int\limits_{a}^{b} {\int\limits_{a}^{b} { \cdots \int\limits_{a}^{b} {\varphi_{{k_{0} }} (z_{0} )\varphi_{{k_{1} }} (z_{1} ) \cdots \varphi_{{k_{n} }} (z_{n} )f(z_{0} ,z_{1} , \ldots z_{n} )|d{\mathbf{z}}|} } } \approx \\ & \quad \;\sum\limits_{{m_{0} = 0}}^{{\omega_{0} }} {\sum\limits_{{m_{1} = 0}}^{{\omega_{1} }} { \cdots \sum\limits_{{m_{n} = 0}}^{{\omega_{n} }} {L_{{m_{0} ,m_{1} , \ldots ,m_{n} }} } } } \int\limits_{a}^{b} {\int\limits_{a}^{b} { \cdots \int\limits_{a}^{b} {\varphi_{{k_{0} }} (z_{0} )\varphi_{{m_{0} }} (z_{0} ) \cdots \varphi_{{k_{n} }} (z_{n} )\varphi_{{m_{n} }} (z_{n} )|d{\mathbf{z}}|}}}\\&\quad = \sum\limits_{{m_{0} = 0}}^{{\omega_{0} }} {\sum\limits_{{m_{1} = 0}}^{{\omega_{1} }} { \cdots \sum\limits_{{m_{n} = 0}}^{{\omega_{n} }} {L_{{m_{0} ,m_{1} , \ldots ,m_{n} }} } } } \delta_{{m_{0} k_{0} }} \delta_{{m_{1} k_{1} }} \cdots \delta_{{m_{n} k_{n} }} = L_{{k_{0} ,k_{1} , \ldots ,k_{n} }} ,\forall k_{0} ,k_{1} , \ldots ,k_{n} = 0 \ldots \omega, \\ \end{aligned} $$

where $|{\text{d}}{\mathbf{z}}| = {\text{d}}z_{0} {\text{d}}z_{1} \cdots {\text{d}}z_{n}$. By definition

10 $$ E[\varphi_{{k_{0} }} (z_{0} )\varphi_{{k_{1} }} (z_{1} ) \cdots \varphi_{{k_{n} }} (z_{n} )] = \int\limits_{a}^{b} {\int\limits_{a}^{b} { \cdots \int\limits_{a}^{b} {\varphi_{{k_{0} }} (z_{0} )\varphi_{{k_{1} }} (z_{1} ) \cdots \varphi_{{k_{n} }} (z_{n} )f(z_{0} ,z_{1} , \ldots z_{n} )|{\text{d}}{\mathbf{z}}|} } }. $$

When considering the spatial locations ${\mathbf{u}} = \{ {\mathbf{u}}_{0} ,{\mathbf{u}}_{1} , \ldots ,{\mathbf{u}}_{n} \}$ of random variables $Z_{0} ,Z_{1} , \ldots Z_{n}$, the coefficients $L_{{k_{0} ,k_{1} , \ldots ,k_{n} }}$ can be estimated from available data using Eqs. (9) and (10) by calculating

11 $$ L_{{k_{0} ,k_{1} , \ldots k_{n} }} \approx E[\varphi_{{k_{0} }} (z_{0} )\varphi_{{k_{1} }} (z_{1} ) \cdots \varphi_{{k_{n} }} (z_{n} )] \approx \frac{1}{{N_{{h_{1} ,h_{2} , \ldots h_{n} }} }}\sum\limits_{k = 1}^{{N_{{h_{1} ,h_{2} , \ldots h_{n} }} }} {\varphi_{{k_{0} }} (z_{0}^{k} )\varphi_{{k_{1} }} (z_{1}^{k} ) \cdots \varphi_{{k_{n} }} (z_{n}^{k} )}, $$

where values $z_{i}^{k} ,i = 0 \ldots n$ are taken from the available data $z_{i}^{k} \in {\mathbf{d}}_{n}$ and the given training image, and separated by lags ${\mathbf{h}}_{i} = {\mathbf{u}}_{i} - {\mathbf{u}}_{0} ,i = 1 \ldots n$.

Finally, high-order sequential simulations are generated using the following algorithm:

Algorithm A.1

1. Define a random path for visiting all unsampled nodes on the simulation grid.
2. For each node **u**_0_ in the path:
  - Find the closest sampled grid nodes ${\mathbf{u}}_{1} ,{\mathbf{u}}_{2} , \ldots {\mathbf{u}}_{n}$.
  - Calculate lags ${\mathbf{h}}_{i} = {\mathbf{u}}_{i} - {\mathbf{u}}_{0} ,i = 1 \ldots n$ for unsampled location **u**_0_.
  - Scan the initial data and find values $z_{k}^{i} ,i = 0 \ldots n$ separated by lags ${\mathbf{h}}_{i} = {\mathbf{u}}_{i} - {\mathbf{u}}_{0} ,i = 1 \ldots n$.
  - Calculate the coefficients $L_{{k_{0} ,k_{1} , \ldots ,k_{n} }}$ using Eq. (11).
  - Build the cpdf $f({\mathbf{u}}_{0} ;z_{0} |z_{1} , \ldots z_{n} )$ for the random variable *Z*_0_ at the unsampled location **u**_0_ given the conditioning data $z_{1} , \ldots z_{n}$ at the corresponding neighbors ${\mathbf{u}}_{1} ,{\mathbf{u}}_{2} , \ldots {\mathbf{u}}_{n}$ using Eqs. (2) and (8).
  - Draw a uniform random value in [0, 1] to generate a simulated value *z*_0_ from the conditional distribution $f({\mathbf{u}}_{0} ;z_{0} |z_{1} , \ldots z_{n} )$.
  - Add *z*_0_ to the set of sample data and the previously simulated values.
3. Repeat Steps 2a–g for the next points in the random path defined in Step 1.

### Legendre Polynomials

Mustapha and Dimitrakopoulos (2010) proposed using a Legendre series as a set of basis functions $\varphi_{1} (z),\varphi_{2} (z), \ldots$. The Legendre polynomial *P*_*k*_ of order *k* is defined as in Lebedev (1965)

12 $$ P_{k} = \frac{1}{{2^{k} k!}}\left( {\frac{{{\text{d}}^{k} }}{{{\text{d}}z^{k} }}} \right)\left[ {(z^{2} - 1)^{k} } \right],\quad - 1 \le z \le 1. $$

The set of Legendre polynomials $\{ P_{k} (z)\}_{k}$ forms a complete basis set on the interval [− 1, 1], and, accordingly, the function $f({\mathbf{u}}_{0} ;z_{0} |z_{1} , \ldots z_{n} )$ can be approximated using Eqs. (2) and (8). By their construction, the order of Legendre polynomials corresponds to the order of high-order spatial statistics of the probability function $f({\mathbf{u}}_{0} ;z_{0} |z_{1} , \ldots z_{n} )$. However, there are practical limitations when using Legendre polynomials for the approximation of functions in multidimensional space. This is discussed in Sect. 3.

### Legendre-Like Orthogonal Splines Approximation

In the present work, Legendre-like splines (Wei et al. 2013) are used as a set of basis functions. These splines are constructed using Legendre polynomials and the linear combination of B-splines. B-splines of order *r* in a variable $t \in [a,b]$ are piecewise polynomials defined over the domain

13 $$ T = \{ \underbrace {{a,a, \ldots ,t_{0} = a}}_{r + 1} < t_{1} \le t_{2} \le \ldots \le t_{{m_{\hbox{max} } }} < \underbrace {{t_{{m_{\hbox{max} } + 1}} = b,b, \ldots ,b}}_{r + 1}\} . $$

The points $t_{i} ,i = 0 \ldots m_{\hbox{max} }$ are called knots. Each piece, a B-spline of order *r*, can be derived using de Boor’s formula (de Boor 1978)

14 $$ B_{i,0} = \left\{ {\begin{array}{*{20}l} {1,} \hfill & {t_{i} \le t \le t_{i + 1} } \hfill \\ {0,} \hfill & {\text{otherwise}} \hfill \\ \end{array} } \right. $$

15 $$ B_{i,r} (t) = \frac{{t - t_{i} }}{{t_{i + r - 1} - t_{i} }}B_{i,r - 1} (t) + \frac{{t_{i + r} - t}}{{t_{i + r} - t_{i + 1} }}B_{i + 1,r - 1} (t). $$

B-splines do not form an orthogonal basis; however, Wei et al. (2013) introduced orthogonal splines based on the combination of B-splines and a set of knot sequences.

The first *r* + 1 splines are defined as the Legendre polynomials up to order *r*

16 $$ S_{k} (t) = P_{k} (t),k = 0 \ldots r. $$

The subsequent splines are constructed on subsets $T_{m} = \{ t_{i,m} \}_{i = - r}^{r + m + 1}$, $m = 1 \ldots m_{\hbox{max} } - 1$ of the knot sequence *T*, where the *t*_*i*,*m*_ are defined as follows

17 $$ t_{i,m} = \left\{ {\begin{array}{*{20}l} {a,} \hfill & { - r \le i \le 0} \hfill \\ {t_{i} ,} \hfill & {1 \le i \le m} \hfill \\ {b,} \hfill & {m + 1 \le i \le m + r + 1}. \hfill \\ \end{array} } \right. $$

For example, the first and second subsets are $T_{1} = \{ \underbrace {a,a, \ldots ,a}_{r + 1} < t_{1} < \underbrace {b,b, \ldots ,b}_{r + 1}\}$ and $T_{2} = \{ \underbrace {a,a, \ldots ,a}_{r + 1} < t_{1} \le t_{2} < \underbrace {b,b, \ldots ,b}_{r + 1}\}$, respectively. Let $B_{i,r,m} (t)$ be a B-spline of order *r* on the knot sequence *T*_*m*_

18 $$ B_{i,0,m} = \left\{ {\begin{array}{*{20}l} {1,} \hfill & {t_{i,m} \le t \le t_{i + 1,m} } \hfill \\ {0,} \hfill & {\text{otherwise}} \hfill \\ \end{array} } \right. $$

19 $$ B_{i,r,m} (t) = \frac{{t - t_{i,m} }}{{t_{i + r - 1,m} - t_{i,m} }}B_{i,r - 1,m} (t) + \frac{{t_{i + r,m} - t}}{{t_{i + r,m} - t_{i + 1,m} }}B_{i + 1,r - 1,m} (t), $$

then, the remaining Legendre-like splines $S_{k} (t),k = r + 2 \ldots r + m_{\hbox{max} }$ are determined by

20 $$ S_{r + m} (t) = \frac{{d^{r + 1} }}{{dt^{r + 1} }}f_{m} (t),m = 1 \ldots m_{\hbox{max} }, $$

where *f*_*m*_(*t*) is the determinant of the matrix:

21 $$ f_{m} (t) = \det \left( {\begin{array}{*{20}c} {B_{ - r,2r + 1,m} (t)} & {B_{ - r + 1,2r + 1,m} (t)} & \cdots & {B_{ - r + m - 1,2r + 1,m} (t)} \\ {B_{ - r,2r + 1,m} (t_{1} )} & {B_{ - r + 1,2r + 1,m} (t_{1} )} & \vdots & {B_{ - r + m - 1,2r + 1,m} (t_{1} )} \\ \vdots & \vdots & \ddots & \vdots \\ {B_{ - r,2r + 1,m} (t_{m - 1} )} & {B_{ - r + 1,2r + 1,m} (t_{m - 1} )} & \cdots & {B_{ - r + m - 1,2r + 1,m} (t_{m - 1} )} \\ \end{array} } \right). $$

**Fig. 1:**
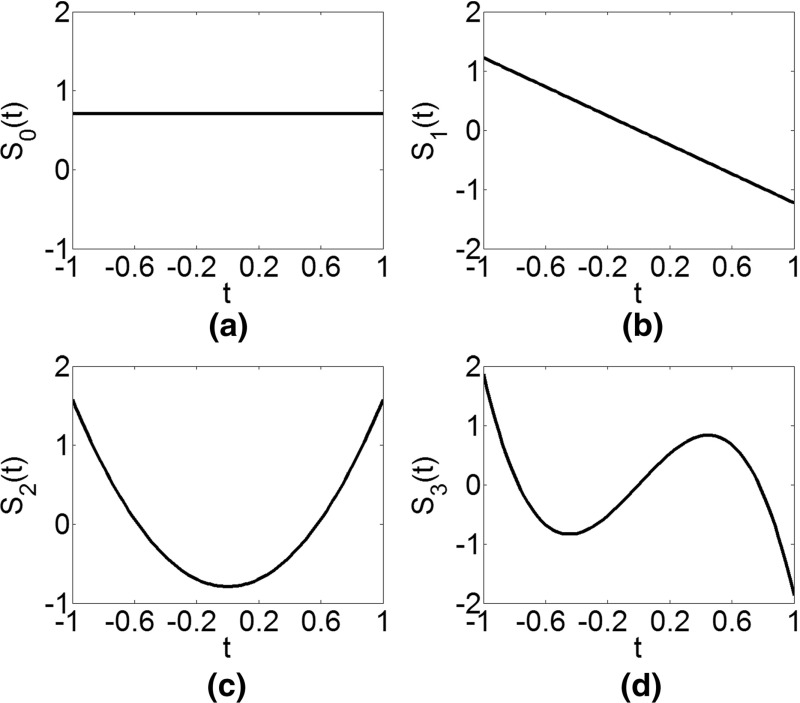
The first four Legendre-like splines over the knot sequence *T*

**Fig. 2:**
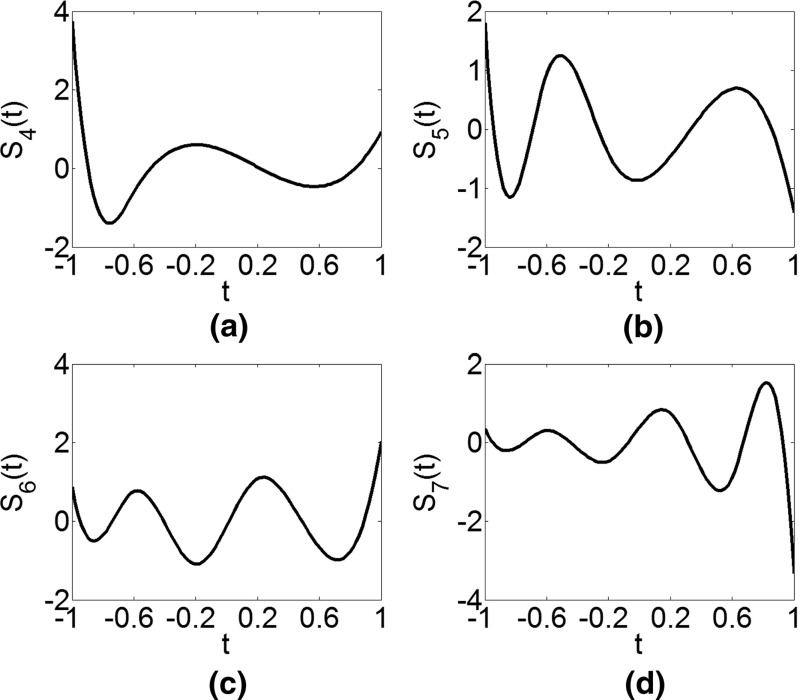
The last four Legendre-like splines over the knot sequence *T*

The examples of orthogonal splines of order *r* = 3 and the knot sequence $T = [ - 1, - 1, - 1, - 1, - 0.6, - 0.2,0.2,0.6,1,1,1,1]$ are presented in Figs. 1 and 2. The first *r* + 1 splines are defined on the knot sequence with only one interval [− 1, 1] and are, thus, simply Legendre polynomials up to the order *r* (Fig. 1). For each subsequent spline, the knot sequence is updated by adding a knot from the initial knot sequence *T*, e.g., the fifth spline (Fig. 2a) is defined by Eq. (20) on two intervals [− 1, − 0.6] and [− 0.6, − 1], or the knot sequence $T_{1} = [ - 1, - 1, - 1, - 1, - 0.6,1,1,1,1]$). It should be noted that Eq. (20) is obtained from the condition of orthogonality in respect to all previous splines. The following three splines (Fig. 2b–d) are defined on the knot sequence $T_{2} = [ - 1, - 1, - 1, - 1, - 0.6, - 0.2,1,1,1,1]$, $T_{3} = [ - 1, - 1, - 1, - 1, - 0.6, - 0.2,0.2,1,1,1,1]$, and *T*_4_ = *T*.

**Fig. 3:**
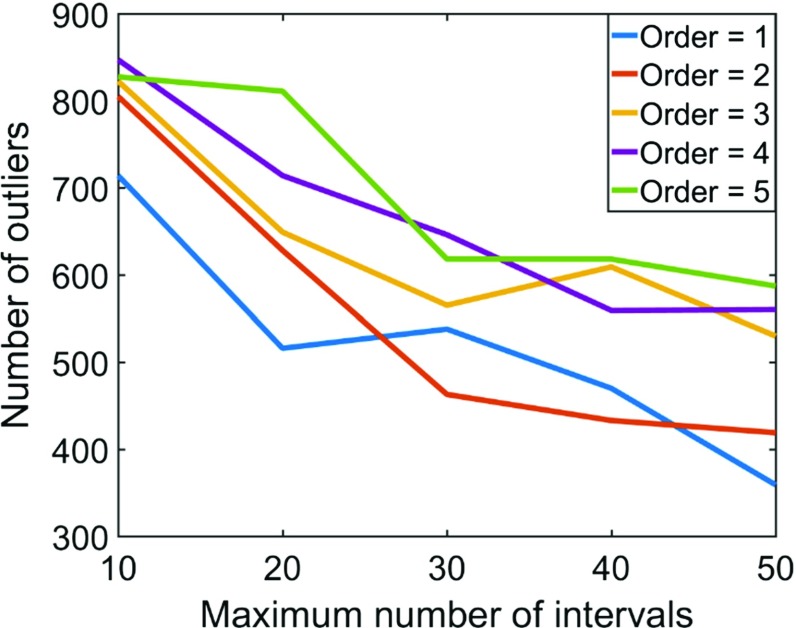
The number of outliers depending on the order of splines *r* and the maximum number of intervals *m*_max_. Low number of outliers corresponds to good quality of approximation

In this work, the initial values are linearly transformed into the [− 1, 1] values range and divided into *m*_max_ intervals. There are two parameters that have a significant impact on the quality of approximation: the maximum number of intervals *m*_max_ and the order of splines *r*. These parameters reflect the maximum order of high-order spatial statistics that can be captured from the available data. The first parameter is the order of splines *r*. High values of the order *r* lead to a non-stable approximation (Runge 1901; Platte et al. 2011) and high computational costs, whereas low values affect the continuity and smoothness of approximation. For example, zero-order splines are good for the approximation of the stepwise function because each spline is a constant function. Splines with *r* = 1 are used for the approximation of continuous, but not smooth, functions, i.e., a polygonal line. In practice, cubic splines, i.e., *r* = 3, are commonly used (Hughes et al. 2005; Piegl 1989). The second parameter is the maximum number of intervals *m*_max_. Low values of *m*_max_ lead to an approximation that is close to a polynomial case, for which limitations are discussed in Sect. 4. For example, an approximation with *m*_max_ = 1 corresponds to the Legendre polynomial approximation presented by Mustapha and Dimitrakopoulos (2010). High values of *m*_max_ result in overfitting or poor predictive performance, as it overreacts to minor fluctuations in the data. In addition, an approximation with a high value of *m*_max_ affects the variability of the simulations because it directly samples values from the initial available data and pastes them into simulations. To choose values of *r* and *m*_max_, different measures are tested. The widely known Kolmogorov–Smirnov statistics test (Stephens 1974) that indicates whether two data samples come from the same distribution is not utilized herein because the related quantile–quantile plot is reproduced well for a very wide range of *r* and *m*_max_ values; thus, such a statistical test does not provide guidance on selecting suitable parameters. Other approaches, including comparing high-order spatial statistics maps or connectivity properties, are hard to quantify by a single number and complex to implement. In this work, a simple and fast measure of the quality of approximation is used. This quality of approximation is expressed in terms of the number of grid nodes where splines fail to approximate the conditional distribution. At these nodes, the high-order simulation method produces numerical artefacts, such as outliers or noise values. Outliers can be easily detected by comparing the value at nodes with their local neighborhood average value. The average number of outliers is calculated for different values of *r* and *m*_max_ (Fig. 3). According to Fig. 3, as the number of intervals *m*_max_ is increased, the quality of approximation improves. At the same time, increasing the order of splines *r* decreases the quality of approximation. For cubic splines (order *r* = 3), the reasonable number of intervals is 30, as it provides the same quality of approximation as 50, demonstrates better predictive performance, and is computationally less expensive. The corresponding order of high-order spatial statistics is *m*_max_ + *r* = 33.

## Testing the Simulation Approach with a Fully Known Dataset

**Fig. 4:**
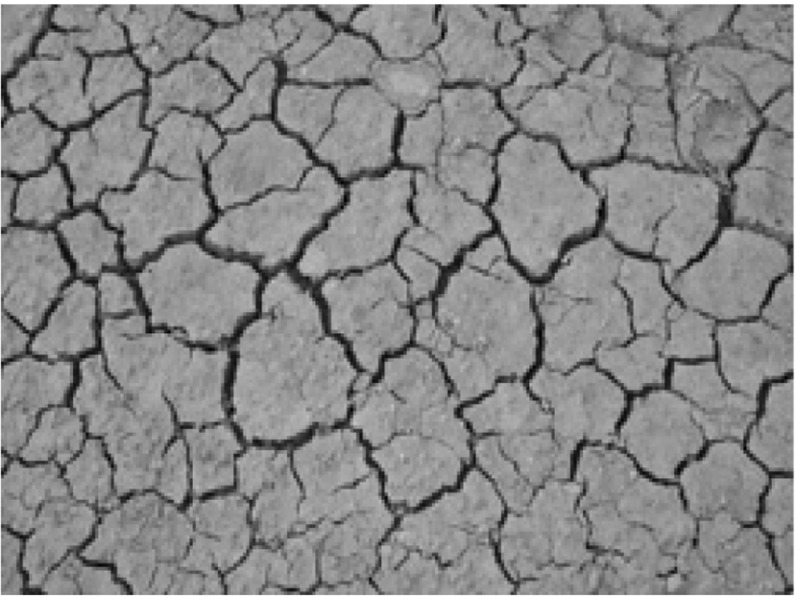
Image of a fracture network (public dataset)

**Fig. 5:**
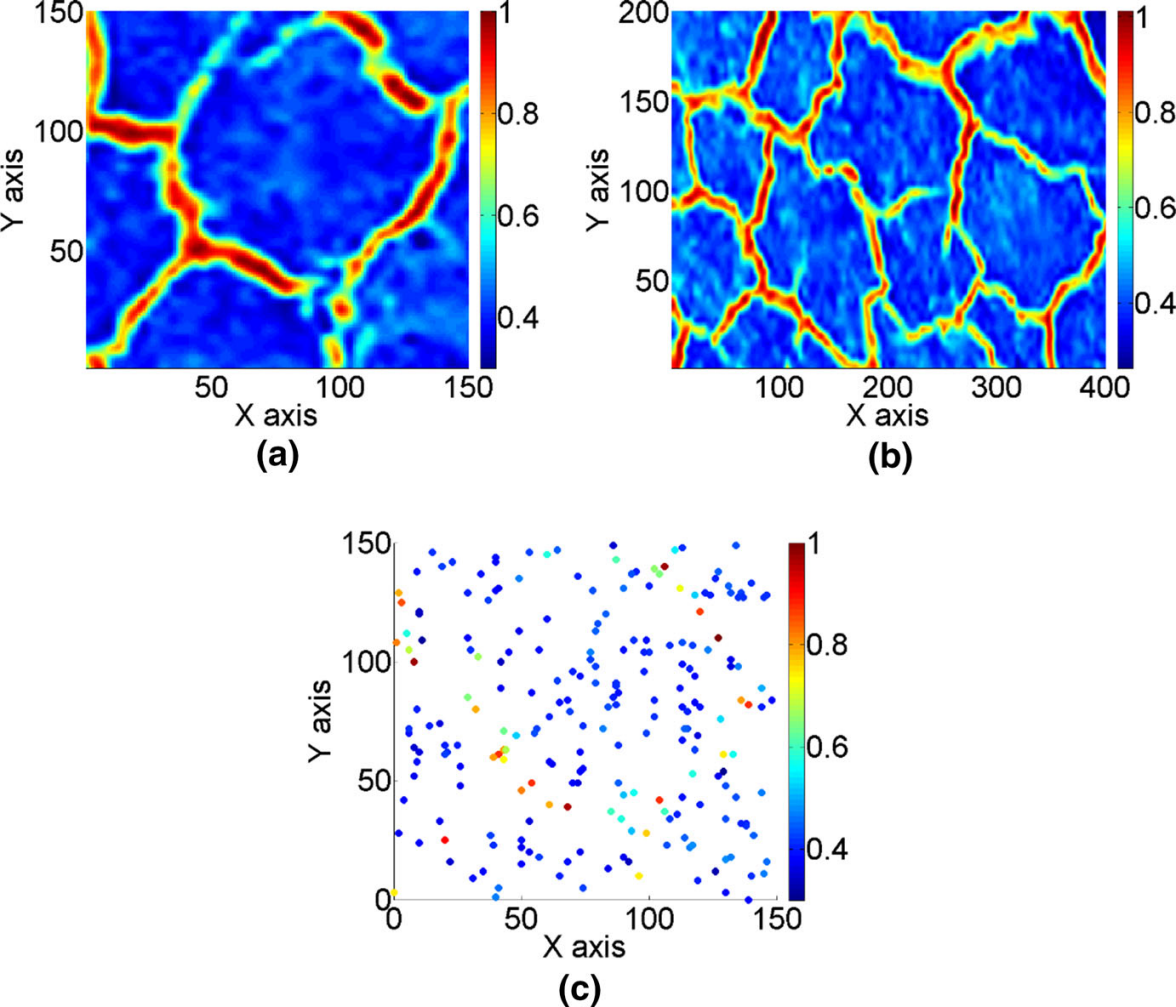
Fracture network: **a** reference image; **b** training image; **c** dataset from reference image

The high-order simulation method presented in the previous section is tested with a fully known dataset obtained from an image of a fracture network downloaded from a texture synthesis website (http://br.depositphotos.com/5211338/stock-photo-dry-terrain.html) and displayed in Fig. 4. Gray-scale values of the image are transformed to the [0, 1] domain. The reference image (Fig. 5a) and the TI (Fig. 5b) are taken from different parts of the image and have sizes 150 × 150 and 200 × 400 grid nodes, respectively. The dataset (Fig. 5c) is generated from the reference image with a uniform random sampling of 225 values (1% of the image points).

**Fig. 6:**
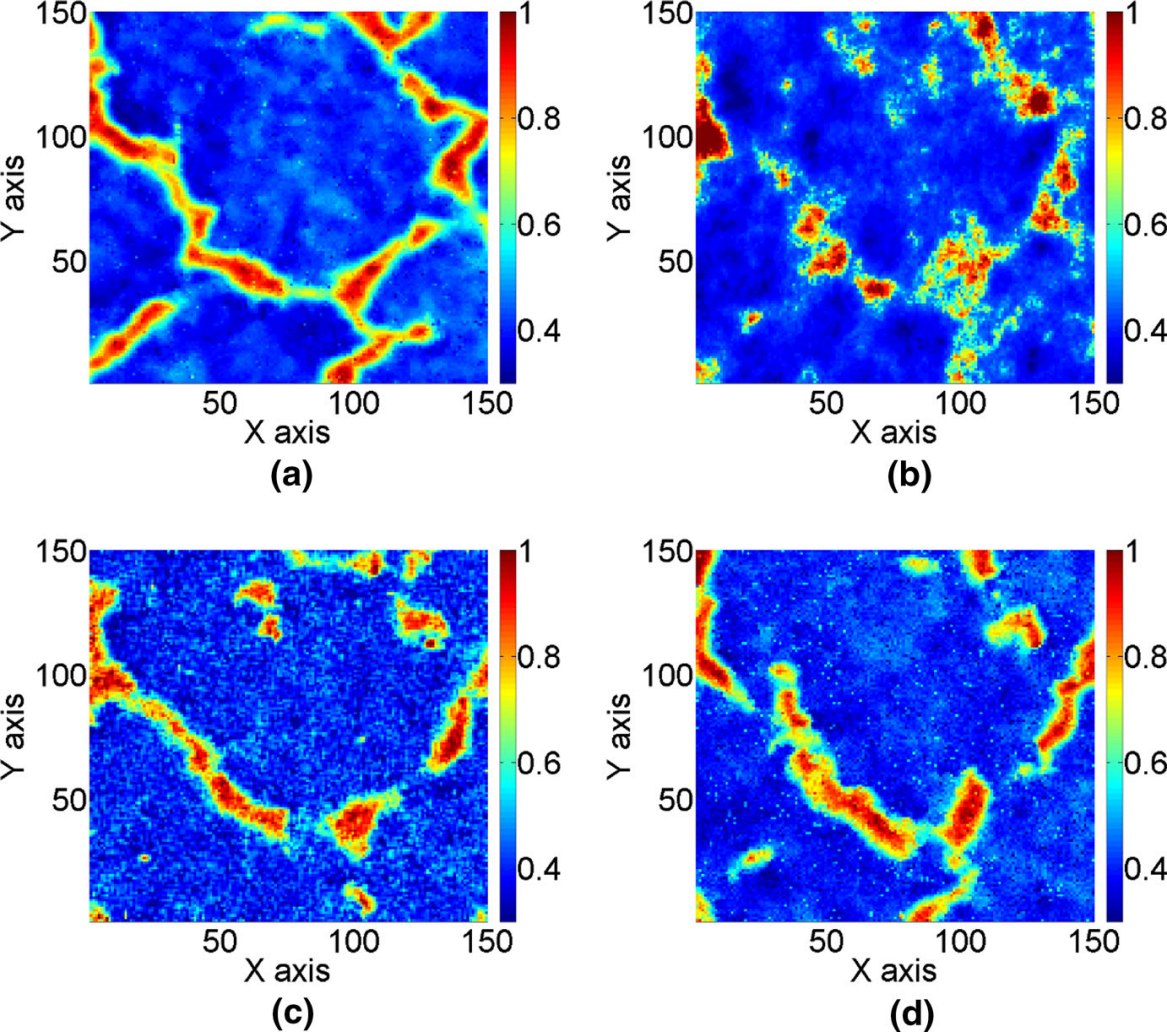
The simulation results are as follows: **a** the simulation using *hosim*-*splines*, **b** the simulation using *sgsim*, and **c**, **d** the simulations using *hosim*-*polynomials* of orders 10 and 20, respectively

Three different systems of functions for the high-order simulation approach (*hosim*) and the sequential Gaussian simulation (*sgsim*) method are compared next: (a) Legendre-like splines (*r* = 3, *m*_max_ = 30, the corresponding order of high-order spatial statistics is *m*_max_ + *r* = 33), (b) *sgsim* method, (c) Legendre polynomials of order 10 (the corresponding order of high-order spatial statistics is 10), and (d) Legendre polynomials of order 20 (the corresponding order of high-order spatial statistics is 20). Hereafter, the system of functions based on splines and Legendre polynomials are correspondingly called *hosim*-*splines* and *hosim*-*polynomials*. The simulation using *hosim*-*splines* (Fig. 6a) shows a stable reproduction of spatially connected structures. Simulations using *hosim*-*polynomials* (Fig. 6c, d) have less connected features than the simulation using splines. *sgsim* (Fig. 6b) fails to reproduce the spatial continuity of high values. Table 1 shows the average value, median, and variance for the sample data, reference image, TI, and simulated realizations; note that only *hosim*-*polynomials* of order 20 are included in the comparisons that follow. All methods reproduce well the low-order statistics of the sample data and the TI.

**Table 1:** The basic statistics of the sample data, reference image, training image, and simulations

Data and simulations	Average	Median	Variance
The sample data	0.47	0.41	0.021
The reference image	0.48	0.42	0.022
The training image	0.48	0.42	0.023
*hosim*-*splines*	0.48	0.42	0.020
*hosim*-*polynomials*	0.46	0.41	0.018
*sgsim*	0.46	0.41	0.019

**Fig. 7:**
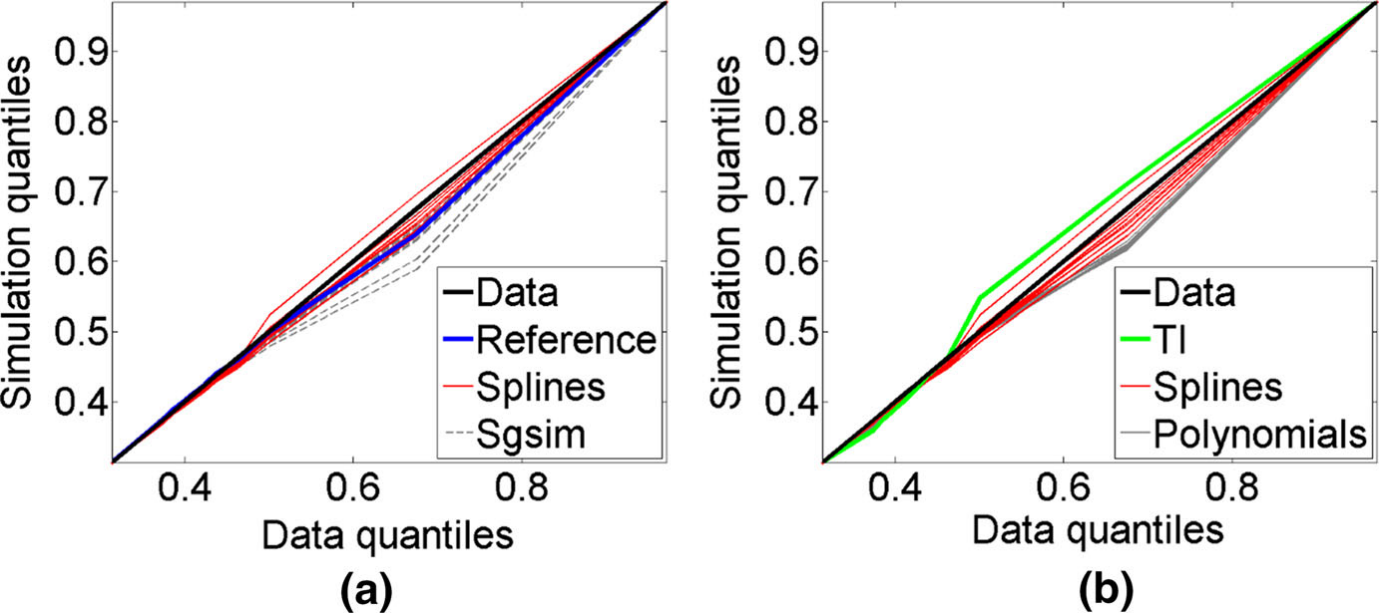
Quantile–quantile (QQ) plots of simulations with the sample data. The blue line is the reference image; the green line is the training image; the red lines are the simulations using splines; the gray solid lines are the simulations using Legendre polynomials; and the gray dashed lines are the simulations using *sgsim*

Figure 7 shows the quantile–quantile (QQ) plots of ten simulated realizations of each simulation approach with the sample data. The QQ plots for the simulations using *hosim*-*splines* are represented by red lines. The 45° black line represent QQ plots of the sample data with the sample data. The blue line represents the QQ plot of the reference image with the sample data. The green line represents the QQ plot of the training image with the sample data. The QQ plots for the simulations using *sgsim* are depicted by gray dashed lines and the QQ plots for the simulations using *hosim*-*polynomials* are shown by gray solid lines. Overall, the QQ plots of simulations using *hosim*-*splines* are consistent with the QQ plot of the sample data and the reference image, whereas QQ plots of simulations using *hosim*-*polynomials* and *sgsim* slightly deviate from the QQ plot of the sample data.

**Fig. 8:**
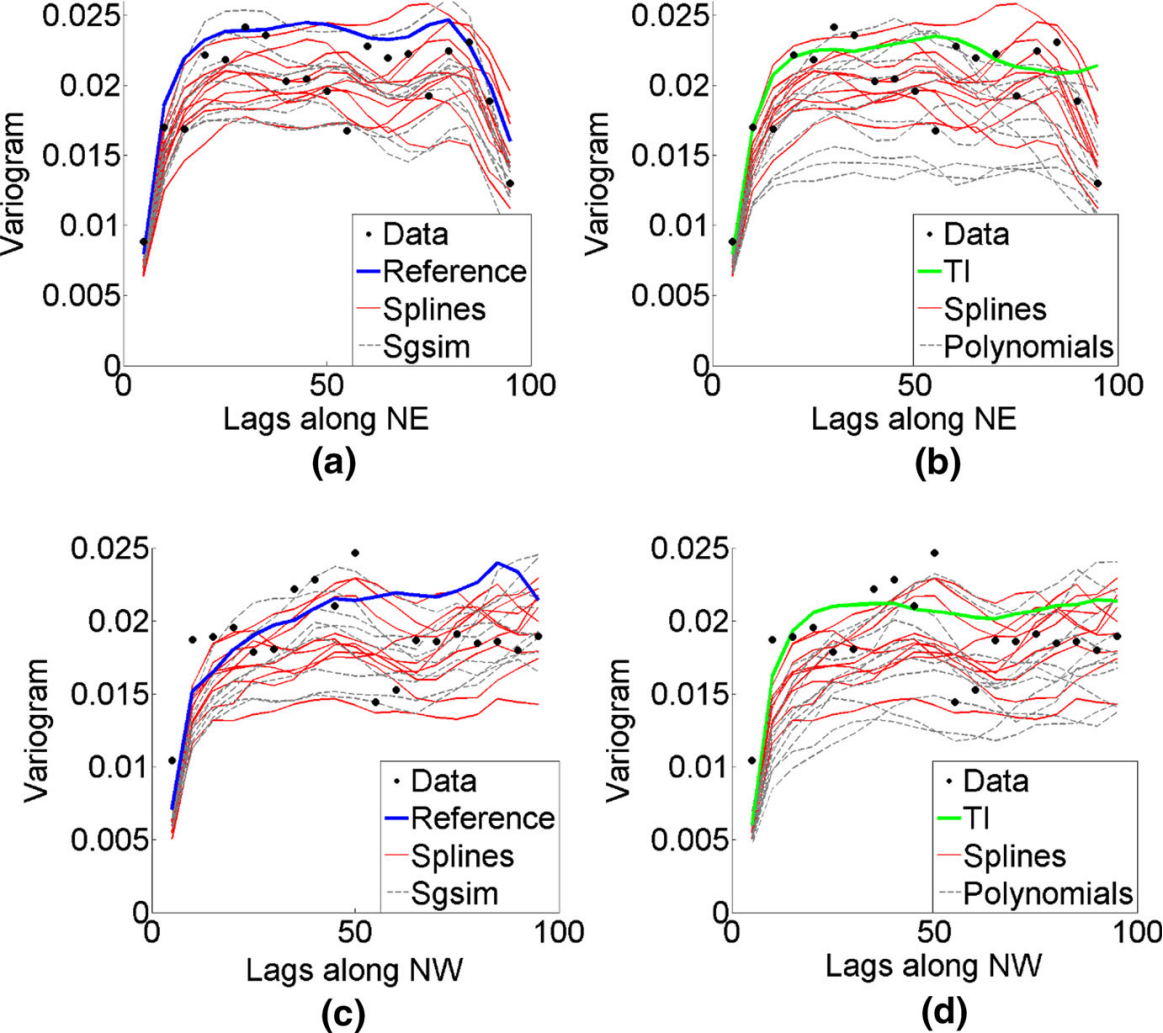
Variograms along the north–east direction (top subfigures **a**, **b**) and north–west direction (bottom subfigures **c**, **d**) of the sample data (dots), reference image (blue line), training image (green line), simulations using *hosim*-*splines* (red lines), simulations using *hosim*-*polynomials* (gray lines), and simulations using *sgsim* (gray dashed lines)

Figure 8 shows variograms along the north–east and north–west directions; that is, directions of the main continuity of high values, calculated for the simulations using *hosim*-*splines* (the red lines), the sample data (dots), the reference image (the blue line), the TI (the green line), the simulations using *hosim*-*polynomials* (the gray solid lines), and the simulations using *sgsim* (the gray dashed lines). All techniques demonstrate reasonable reproduction of the second-order statistics of the sample data.

**Fig. 9:**
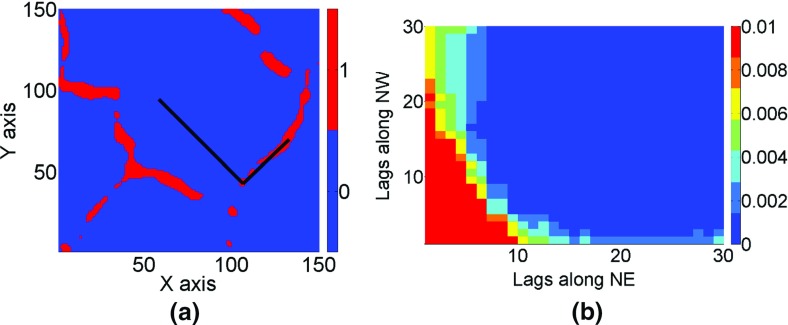
The third-order indicator moment calculation for the reference image: **a** the binary values of the reference image for a cut-off value of 0.82 (95th percentile), the black lines represent L-type template for calculation of the third-order spatial statistics; **b** the third-order spatial moments of the binary image

For the calculation of the third-order and fourth-order spatial statistics, the estimations of the high-order moment are used (Dimitrakopoulos et al. 2010)

22 $$ m_{3} ({\mathbf{h}}_{{\mathbf{1}}} ,{\mathbf{h}}_{{\mathbf{2}}} ) = \frac{1}{{N_{{h_{1} h_{2} }} }}\sum\limits_{i = 0}^{{N_{{h_{1} h_{2} }} }} {Z({\mathbf{u}})Z({\mathbf{u}} + {\mathbf{h}}_{1} )} Z({\mathbf{u}} + {\mathbf{h}}_{2} ), $$

23 $$ m_{4} ({\mathbf{h}}_{1} ,{\mathbf{h}}_{2} ,{\mathbf{h}}_{3} ) = \frac{1}{{N_{{h_{1} h_{2} h_{3} }} }}\sum\limits_{i = 0}^{{N_{{h_{1} h_{2} h_{3} }} }} {Z({\mathbf{u}})Z({\mathbf{u}} + {\mathbf{h}}_{1} )} Z({\mathbf{u}} + {\mathbf{h}}_{2} )Z({\mathbf{u}} + {\mathbf{h}}_{3} ), $$

where *N*_*h*1*h*2_ is the number of elements of replicates found on lags **h**_1_ and **h**_2_, and *N*_*h*1*h*2*h*3_ is the number of elements of replicates found on distances **h**_1_, **h**_2_, and **h**_3_. To highlight the connectivity property along the north–east (NE) and north–west (NW) directions, the third-order moments are calculated for binary images with a cut-off value of 0.82 (95th percentile). An example of a binary image is shown in Fig. 9a. The third-order spatial statistics are estimated based on a template with directional vectors along the NE and NW directions (Fig. 9b), i.e., ${\mathbf{h}}_{1} = (i{\text{d}}x,i{\text{d}}y)$ and ${\mathbf{h}}_{2} = ( - j{\text{d}}x,j{\text{d}}y)$, respectively, where $i,j = 1 \ldots 30$ and the lag discretization along *x* and *y* is d*x* = d*y* = 1 pixel. The physical meaning of the third-order moment of the binary image is straightforward—it is the probability of having high values at the three points separated by lags **h**_1_ and **h**_2_ (Minniakhmetov and Dimitrakopoulos 2017b). The red–orange values represent the average sizes of connected high values along the NE and NW directions. In the third-order indicator moment map of the reference image, the average sizes of the interconnected high values are 10 and 20 pixels along the NE and NW directions, respectively.

**Fig. 10:**
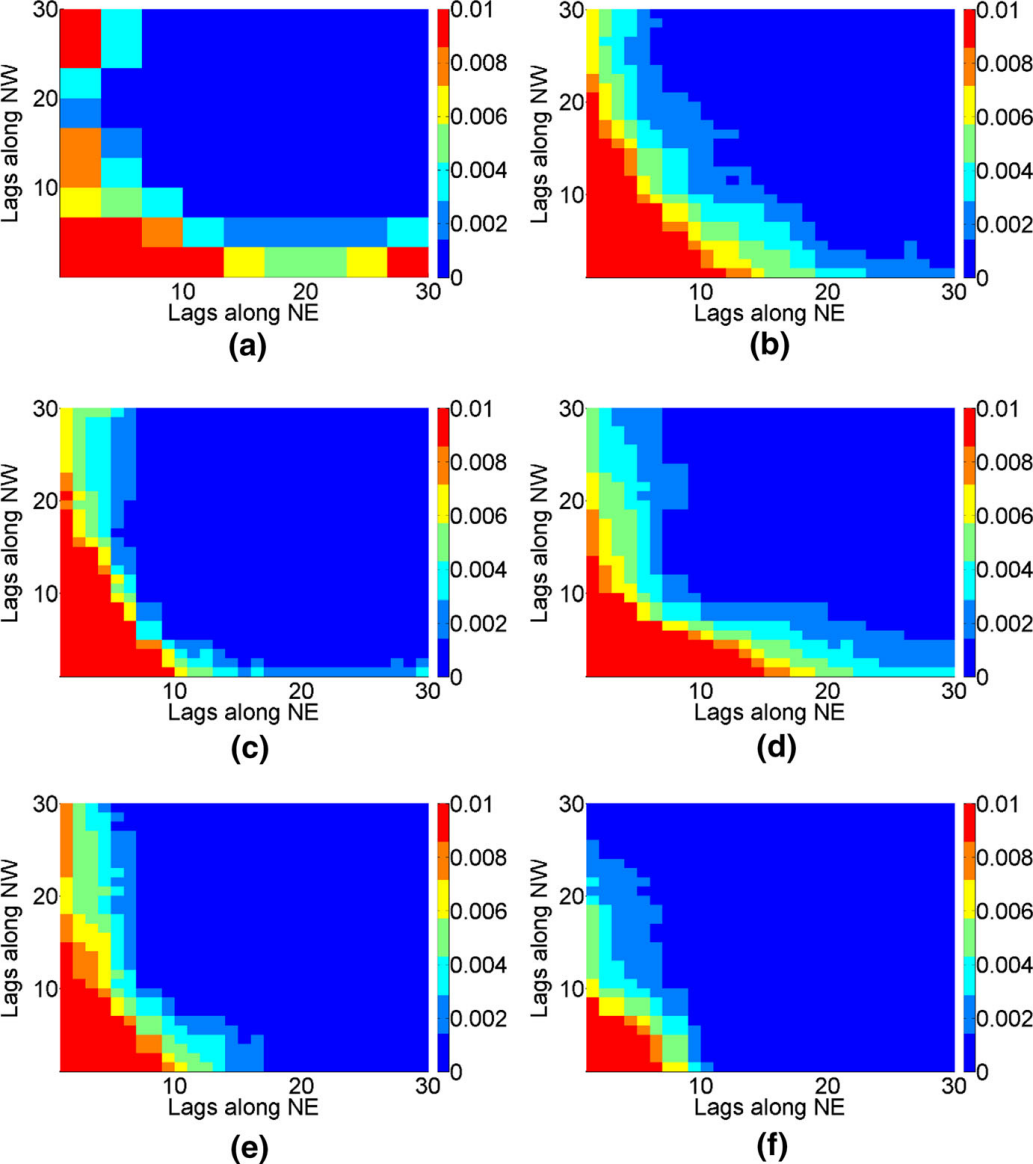
The third-order indicator moments: **a** sample data samples, **b** the simulation using *hosim*-*splines*, **c** the reference image, **d** the training image, **e** a simulation using *hosim*-*polynomials*, and **f** a simulation using *sgsim*

The third-order moments are calculated for simulations and averaged to account for differences between the realizations. The high-order simulation technique using *hosim*-*splines* and *hosim*-*polynomials* (Fig. 10b, e) reproduce the third-order moment map of the sample data (Fig. 10a), the reference image (Fig. 10c), and the TI (Fig. 10d), as can be seen from the similar size of the red–orange value areas in the corresponding figures. The moment map of the simulation using *sgsim* (Fig. 10f) does not reproduce connectivity along the NE and NW directions; the size of the red-shaded area is 8 × 10 pixels, compared to a 10 × 20-pixel area in the reference image’s moment map (Fig. 10c).

**Fig. 11:**
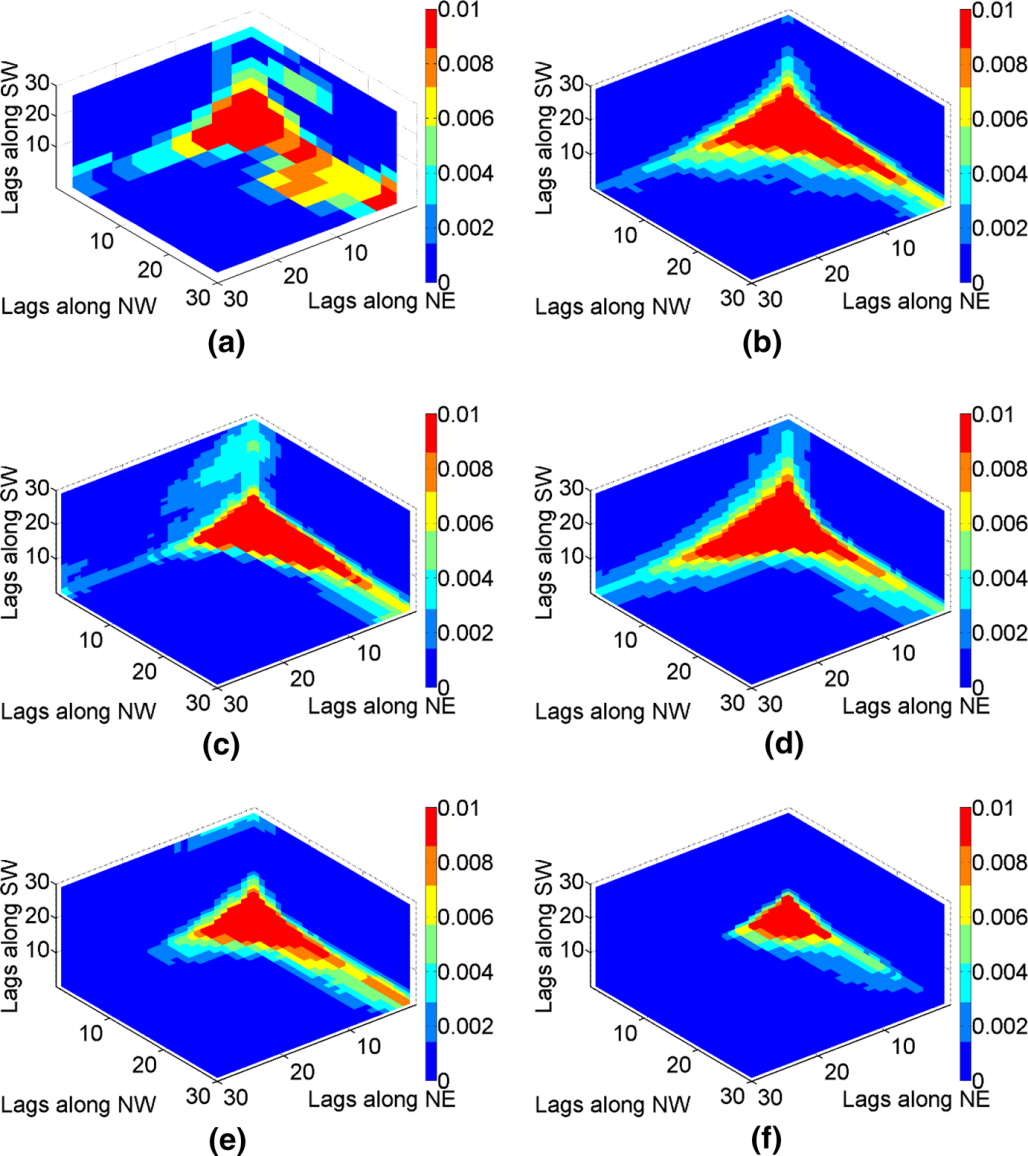
The fourth-order indicator moments: **a** sample data samples, **b** the simulation using *hosim*-*splines*, **c** the reference image, **d** the training image, **e** a simulation using *hosim*-*polynomials*, and **f** a simulation using *sgsim*

The fourth-order spatial statistics are estimated for binary images with a cut-off value of 0.82 (95th percentile) based on a template with directional vectors NE ${\mathbf{h}}_{1} = (i{\text{d}}x,i{\text{d}}y)$, NW ${\mathbf{h}}_{2} = ( - j{\text{d}}x,j{\text{d}}y)$, and south-west (SW) ${\mathbf{h}}_{3} = ( - k{\text{d}}x, - k{\text{d}}y)$, where $i,j,k = 1 \ldots 30$ and lag discretization along *x* and *y* is d*x* = d*y* = 1 pixels. Similarly to the third order, the fourth-order moments are calculated for simulations and averaged to account for differences in the various realizations. The red–orange areas along the axes of the fourth-order spatial statistics (Fig. 11) represent the high values along the NE, NW, and SW directions. According to Fig. 11, the fourth-order moment map for the simulation using *hosim*-*splines* (Fig. 11b) reproduce the sizes of fractures along the NE, NW, and SW directions in the fourth-order moment of the sample data (Fig. 11a), the reference image (Fig. 11c), and the TI (Fig. 11d). The fourth-order moment map of the simulation using *hosim*-*polynomials* (Fig. 11e) shows a smaller connectivity of fractures along the NE and SW directions. The spatial statistics map of the simulation using *sgsim* (Fig. 11f) does not reproduce the connectivity of fractures along the NE and SW directions.

**Fig. 12:**
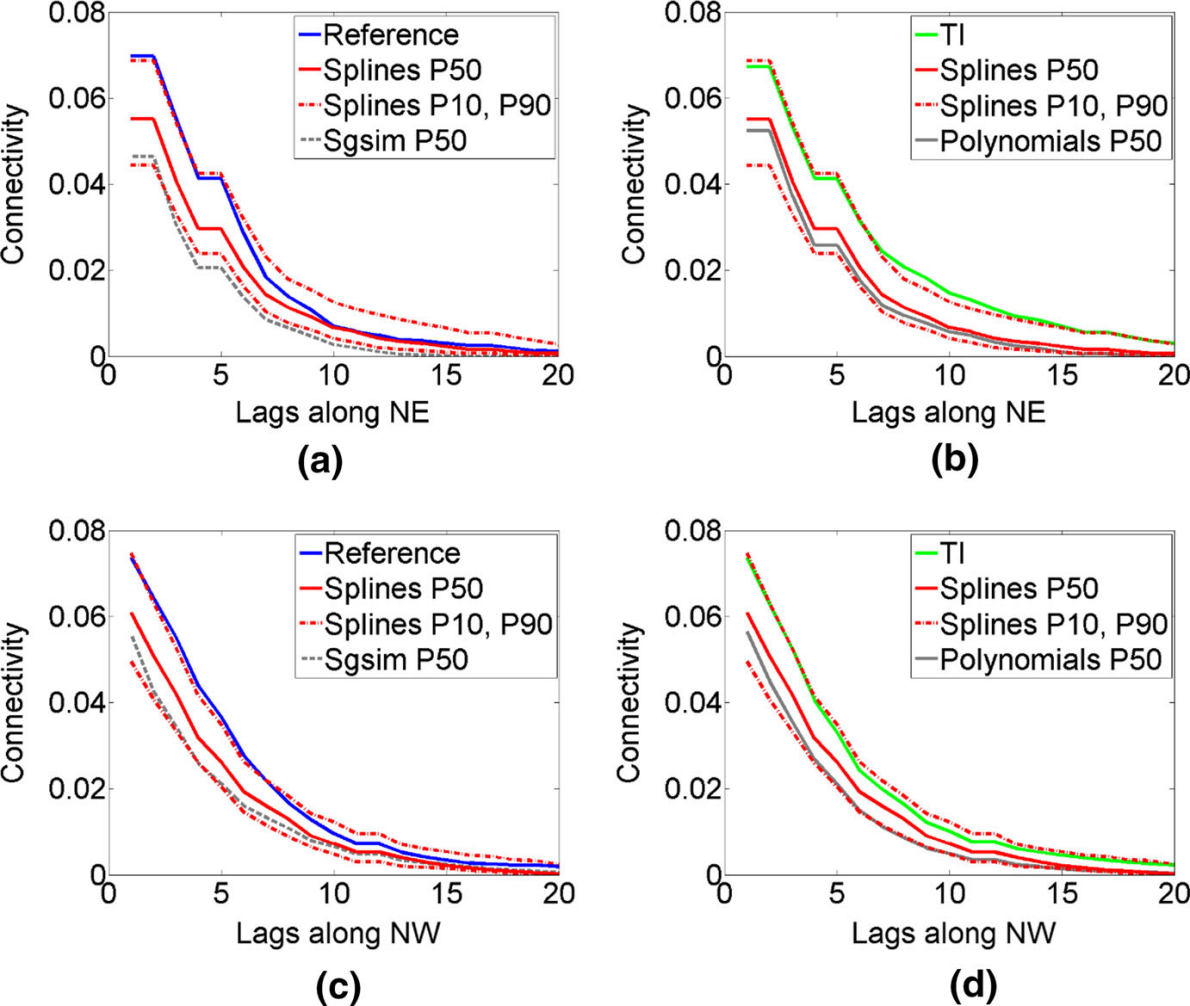
The connectivity functions along the north–east (top subfigures) and north–west directions (bottom subfigures) 95th percentile: reference image (blue line), training image (green line), P50 statistics for simulations using *hosim*-*splines* (red solid line), P10 and P90 statistics for simulations using *hosim*-*splines* (red dash-dot line), P50 statistics for simulations using *hosim*-*polynomials* (gray solid line), and P50 statistics for simulations using *sgsim* (gray dashed line)

The connectivity of high values is measured using the function presented by Journel and Alabert (1989). As in the above examples, the cut-off value is equal to 0.82 (95th percentile). Figure 12 shows P50 statistics of the connectivity measure along the NE (top subfigures) and NW directions (bottom subfigures). The P50 statistics of connectivity are calculated for the simulations using the proposed techniques (red solid line), *hosim*-*polynomials* (gray solid line), and *sgsim* method (gray dashed line). The connectivity measures of the reference image (blue line) and the TI (green line) falls within the P10 and P90 statistics of the connectivity measure in the simulations using *hosim*-*splines* (red dash-dot lines), whereas the connectivity of the simulations using *hosim*-*polynomials* and *sgsim* is lower, on average, than the connectivity of the TI and the reference image.

## Application at a Gold Deposit

Data from a gold deposit are used as a case study to demonstrate the intricacies and advantages of the high-order spatial simulation method described above. In addition, the method is compared with the *sgsim* approach for the reproduction of histograms, variograms, high-order spatial statistics, and the connectivity of high and extreme values.

**Fig. 13:**
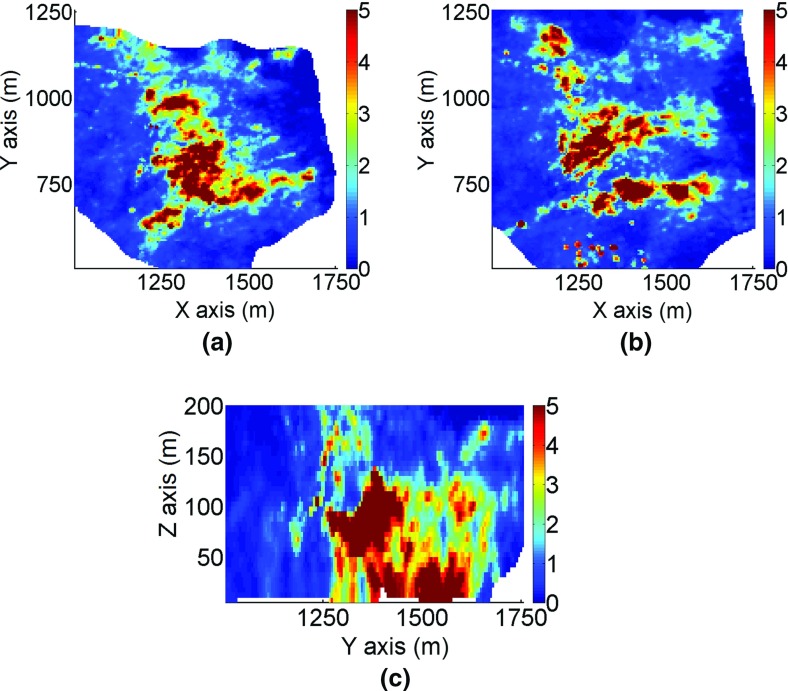
Sections of the training image from part of the deposit: **a** horizontal section at *Z* = 50 m, **b** horizontal section at *Z* = 100 m, and **c** the vertical profile. The colors indicate grades in g/t

**Fig. 14:**
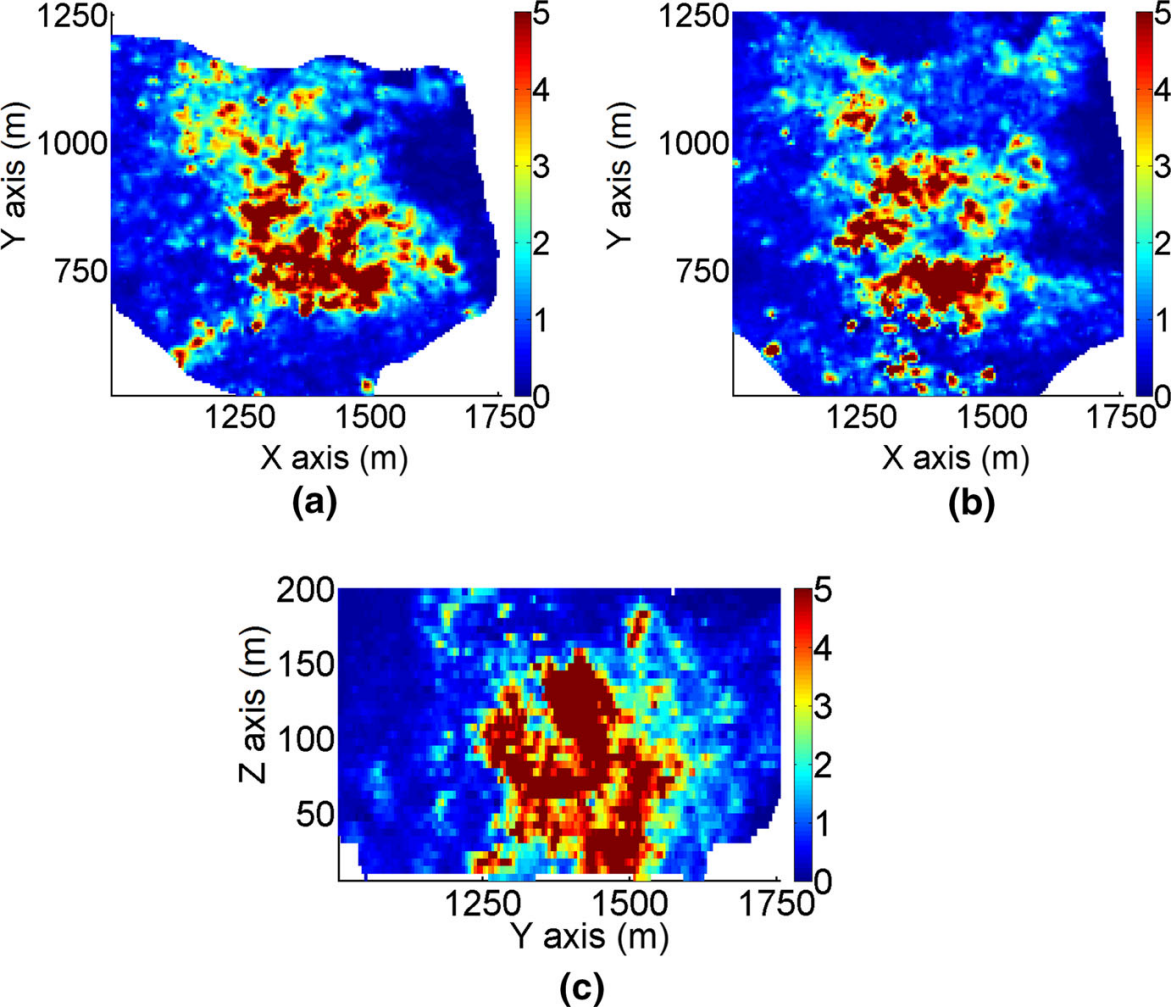
Sections of the simulation using *hosim*-*splines* from part of the deposit: **a** horizontal section at *Z* = 50 m, **b** horizontal section at *Z* = 100 m, and **c** the vertical profile. The colors indicate grades in g/t

The deposit is 2 km by 2 km wide and extends to a depth of 500 m. Sample data are available from 288 exploration drillholes. Blast-hole data are also available for the deposit and used in the construction of a training image. The three-dimensional TI is defined on 405 × 445 × 86 grid blocks of size 5 × 5×5 m^3^. The simulation grid coincides with the grid of the training image. The simulation of grades is performed using the proposed method with cubic splines *r* = 3 and a maximum number of intervals *m*_max_ = 30. Examples of horizontal sections and a vertical profile for the orebody area are shown in Figs. 13 and 14. High grades are located in the south-eastern sector of the deposit, predominantly in the bottom part.

**Fig. 15:**
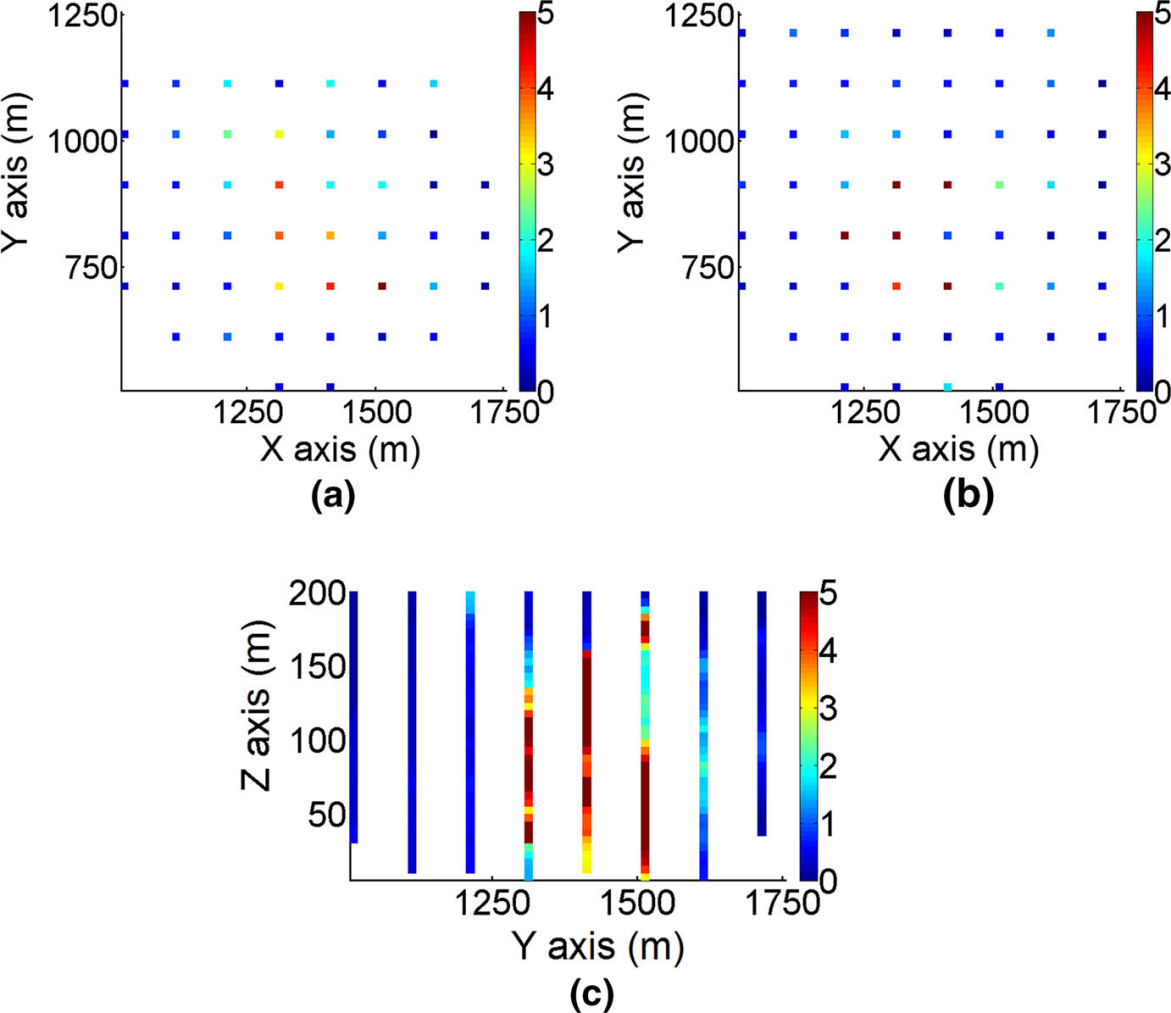
Sections of the exploration drillhole data from part of the deposit: **a** horizontal section at *Z* = 50 m, **b** horizontal section at *Z*= 100 m, and **c** the vertical profile. The colors indicate grades in g/t

**Fig. 16:**
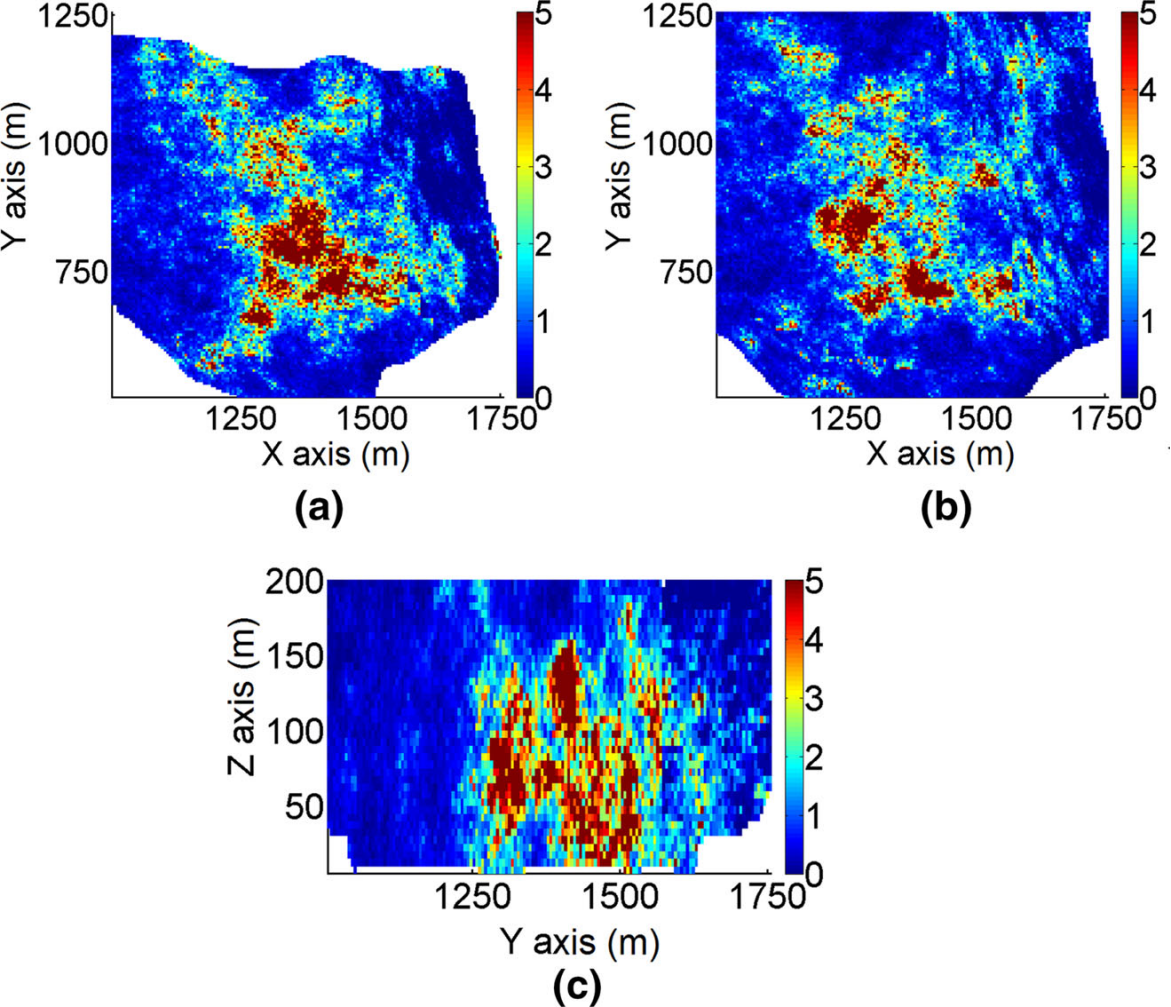
Sections of the simulation using the *sgsim* method from part of the deposit: **a** horizontal section at *Z*= 50 m, **b** horizontal section at *Z* = 100 m, and **c** vertical profile. The colors indicate grades in g/t

The two-dimensional sections in Fig. 14 show that the simulation using the proposed method reproduces the spatial distribution of grades and the continuity of high grades. The areas with high values in Fig. 14 are in good agreement with the drillhole data (Fig. 15) and the TI (Fig. 13). The simulation using *sgsim* (Fig. 16) exhibits a greater number of disconnected structures with high values and sparsely distributed outliers.

These observations are confirmed by a quantitative analysis in a subsequent validation by (1) mean and variance comparison, (2) QQ plots between drillhole data and simulated values, (3) variogram validation, (4) high-order spatial cumulant validation, and (5) connectivity measure. Table 2 shows the average value, median, and variance for the drillhole data, the TI, and the simulations. Both methods reproduce well the low-order statistics of the drillhole data and the TI.

**Table 2:** The basic statistics of the drillhole data, training image, and simulations

Data and simulations	Average	Median	Variance
The drillhole data	0.63	0.38	0.69
The training image	0.66	0.42	0.79
*hosim*	0.65	0.40	0.76
*sgsim*	0.61	0.39	0.67

**Fig. 17:**
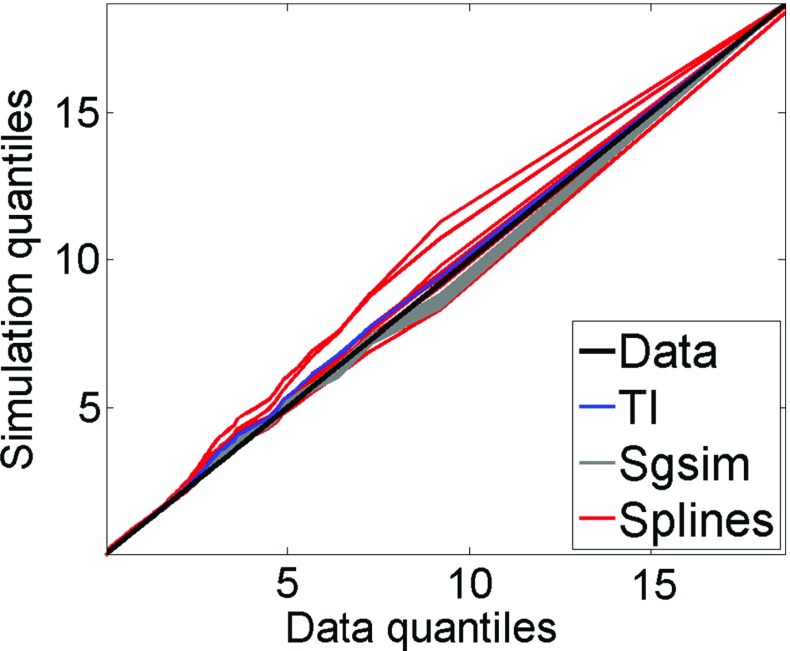
Quantile–quantile (QQ) plots of sample data (black line), training image (TI, blue line), simulations using *hosim*-*splines* (red lines), and simulations using *sgsim* (gray lines)

**Fig. 18:**
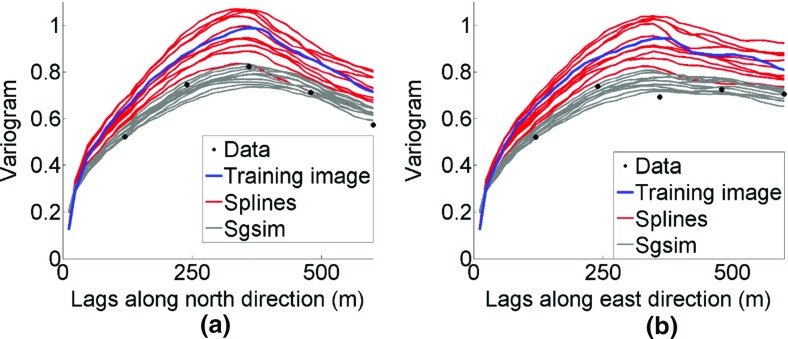
Variograms of the hard data (dots), training image (blue lines), simulations using *hosim*-*splines* (red lines), and simulations using *sgsim* (gray lines) along the north–south (**a**) and east–west (**b**) directions

Figure 17 shows the QQ plots of the simulated realizations and the drillhole data. Quantiles of simulations using *hosim*-*splines* are shown by red lines. Quantiles of simulations using the *sgsim* method are shown by gray lines. In addition, the QQ plots of the training image and the drillhole data are depicted by the blue line. The closer these curves are to the 45° black line in the graph, the better they reproduce the distribution of the drillhole data. Both methods provide simulations consistent with the drillhole data in terms of distributions. Figure 18 presents variograms for the north–south and east–west directions. Simulations using *hosim*-*splines* (red lines) share the second-order statistics of drillhole data and the TI (blue lines). The simulations using the *sgsim* method (gray lines) preserve the second-order statistics of the drillhole data (black dots).

**Fig. 19:**
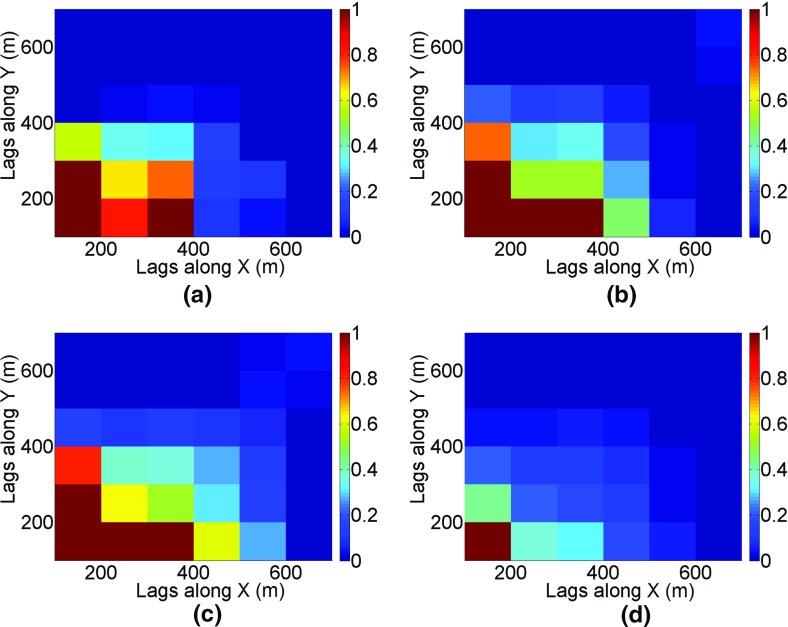
Third-order spatial cumulant maps of the **a** drillhole data, **b** training image (TI), **c** a simulation using *hosim*-*splines*, and **d** a simulation using *sgsim*

Applying a zero-mean transformation, the third-order cumulants can be calculated using Eq. (22) with lags ${\mathbf{h}}_{1} = (i{\text{d}}x,0)\,{\mathbf{h}}_{2} = (0,j{\text{d}}y)$ indexed by $i = 1 \ldots 7,j = 1 \ldots 7$, where d*x* and d*y* are distances between drillholes, that is, 100 m × 100 m. Figure 19 shows the comparison of cumulant maps for sample data, the TI, and the simulations. The values along axes reflect variograms along their corresponding directions because the third-order moment $E(Z^{2} ({\mathbf{x}})Z({\mathbf{x}} + {\mathbf{h}}))$ has similar spatial relations as the second-order moment $E(Z({\mathbf{x}})Z({\mathbf{x}} + {\mathbf{h}}))$. However, the square term *Z*^2^(**x**) in $E(Z^{2} ({\mathbf{x}})Z({\mathbf{x}} + {\mathbf{h}}))$ affects the absolute value of the statistics and, moreover, combines both negative and positive correlations of *Z*(**x**) due to the square operation. Thus, in addition to analyzing the values along the axes, it is important to compare the area of [200; 400] × [200; 400] on the third-order cumulant maps. The simulations using the proposed *hosim*-*splines* method (Fig. 19c) reproduce red areas along the *x*–*y* axes and yellow–green areas in the cumulant map of the drillhole data (Fig. 19a) and the TI (Fig. 19b). These areas reflect the size of connected high grades and are equal to approximately 400 m along the *x*-axis and 300 m along the *y*-axis. The cumulant map for the simulation using *sgsim* (Fig. 19d) neither reproduce the magnitude of the red area along the *x* and *y* axes nor the values at the area of [200; 400] × [200; 400].

**Fig. 20:**
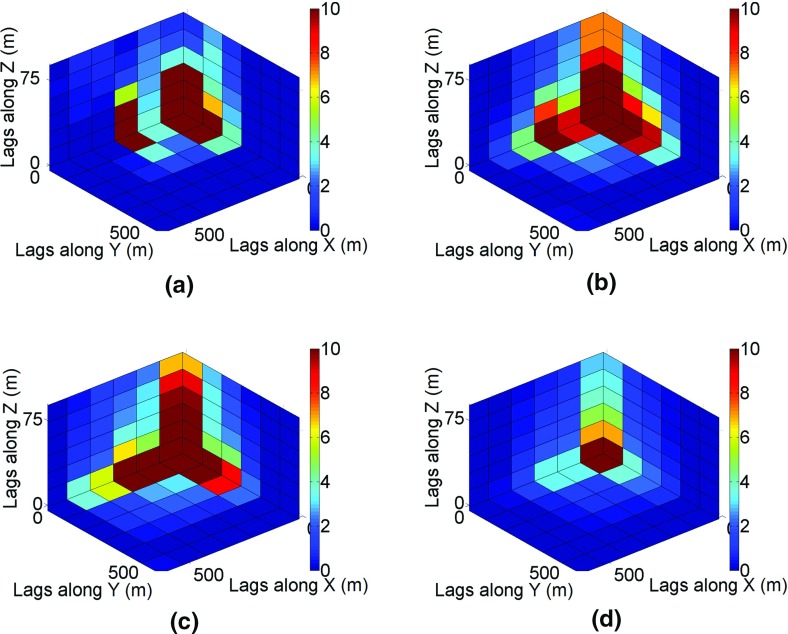
The fourth-order spatial cumulant maps of the **a** drillhole data, **b** training image (TI), **c** a simulation using *hosim*-*splines*, and **d** a simulation using *sgsim*

The fourth-order cumulants are calculated using the following equation

24 $$ \begin{aligned} c_{4} ({\mathbf{h}}_{1} ,{\mathbf{h}}_{2} ,{\mathbf{h}}_{3} ) = \frac{1}{{N_{{h_{1} h_{2} h_{3} }} }}\sum\limits_{i = 0}^{{N_{{h_{1} h_{2} h_{3} }} }} {Z({\mathbf{u}})Z({\mathbf{u}} + {\mathbf{h}}_{1} )} Z({\mathbf{u}} + {\mathbf{h}}_{2} )Z({\mathbf{u}} + {\mathbf{h}}_{3} ) \\ - m_{2} ({\mathbf{h}}_{{\mathbf{1}}} )m_{2} ({\mathbf{h}}_{{\mathbf{2}}} ) - m_{2} ({\mathbf{h}}_{{\mathbf{1}}} )m_{2} ({\mathbf{h}}_{{\mathbf{3}}} ) - m_{2} ({\mathbf{h}}_{{\mathbf{2}}} )m_{2} ({\mathbf{h}}_{{\mathbf{3}}} ), \\ \end{aligned} $$

where *N*_*h*1*h*2*h*3_ is the number of elements of replicates found on distances **h**_1_ and **h**_2_, and *m*_2_(**h**) is the second-order moment along direction **h**, which is equal to the covariance for a zero-mean random field. The lags ${\mathbf{h}}_{1} = (i{\text{d}}x,0),{\mathbf{h}}_{2} = (0,j{\text{d}}y)$, and ${\mathbf{h}}_{3} = (0,k{\text{d}}z)$ are indexed by *i* = 1…7, *j* = 1…7, and *k* = 1…7, where, d*x*, d*y*, and d*z* are distances between data samples, that is, 100 m × 100 m × 5 m. The high-order cumulants calculated reflect the complex structures of orebodies (Dimitrakopoulos et al. 2010). According to Figs. 19 and 20, the size of connected structures is reproduced in simulations using the proposed method (Figs. 19c, 20c). This can also be traced in the vertical profiles (Figs. 13c, 14c, 15c). The fourth-order cumulant map of the simulation using *sgsim* (Fig. 20d) has a rather small red area in comparison with structures in the cumulant maps of the drillhole data (Fig. 20a) and the TI (Fig. 20b).

**Fig. 21:**
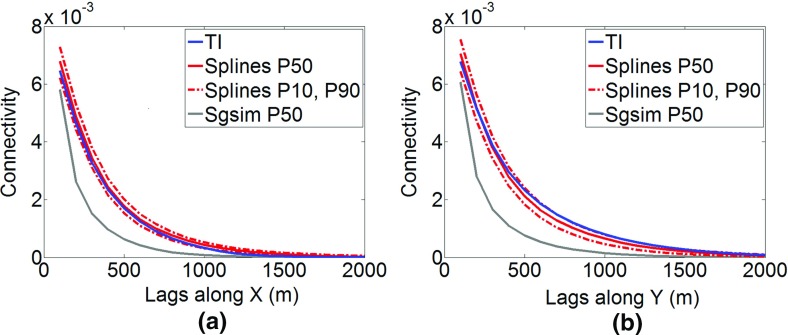
The connectivity along *x* (left subfigure) and *y* (right subfigure) for the 99th percentile: the training image (TI, blue lines), P50 statistics for *hosim* simulations (red solid lines), P10 and P90 statistics for *hosim* simulations (red dashed lines), and P50 statistics for simulations using *sgsim* (gray lines)

The connectivity along the *x* and *y* axes is analyzed using the connectivity measure presented by Journel and Alabert (1989). The cut-off value is equal to 5 ppm (99th percentile). The P10, P50, and P90 statistics of connectivity measures are calculated for simulations using the *hosim*-*splines* method and depicted by red lines in Fig. 21. Solid lines represent the P50 of connectivity measures, whereas dashed lines show the P10 and P90 statistics. The connectivity of the simulations using the proposed method (red lines) remains close to the connectivity measure of the TI (blue lines). The P50 statistics of connectivity measure calculated using *sgsim* simulations (gray lines) is quite far from the connectivity of the TI. Thus, despite reproducing the histograms and variograms, Gaussian simulation methods fail to reproduce an important property of the connectivity of high values.

## Conclusions

This paper presents a novel approach for the high-order simulation of continuous variables based on Legendre-like orthogonal splines. Splines are flexible tools for the approximation of complex probability density functions. Using different knot sequences, orders of splines, and smoothness of piecewise polynomials, it is possible to obtain a stable approximation that reproduces the spatial connectivity of the extreme values. The simulations are consistent with the spatial statistics of the sample data and share the high-order spatial statistics of the available data and the training image.

The proposed approach is also compared with the conventional second-order approach sequential Gaussian simulation and the high-order simulation method using Legendre polynomials. The approach using splines exhibits a more stable approximation of the conditional probability density function (cpdf) and a better representation of the spatial connectivity of extreme values. The applied connectivity measure confirms the results obtained by analyzing the high-order statistics and demonstrates the limitations of Gaussian simulation methods in the characterization of a mineral deposit. In addition, the proposed approach provides a general framework for high-order simulation techniques. For example, by using just one interval for spline construction, the technique reproduces the method proposed by Mustapha and Dimitrakopoulos (2010, 2011).

Further research will address the simulation of categorical variables using splines of order zero and the simulation of multiple correlated continuous and discrete variables within a general framework. In addition, the adaptive knot sequence will be investigated for a better approximation of the cpdf.

## Electronic supplementary material

Below is the link to the electronic supplementary material.

Supplementary material 1 (ZIP 1854 kb)
